# A Prospective Observational Study of Drug Therapy Problems in Pediatric Ward of a Referral Hospital, Northeastern Ethiopia

**DOI:** 10.1155/2020/4323189

**Published:** 2020-03-20

**Authors:** Gizachew Kassahun Bizuneh, Betelhem Anteneh Adamu, Getenet Tadege Bizuayehu, Solomon Debebe Adane

**Affiliations:** ^1^Department of Pharmacognosy, School of Pharmacy, College of Medicine and Health Sciences, University of Gondar, P.O. Box 196, Gondar, Ethiopia; ^2^Department of Pharmacognosy, School of Pharmacy, College of Medicine and Health Sciences, Mizan Tepi University, Mizan, Ethiopia; ^3^Department of Clinical Pharmacy, School of Pharmacy, College of Health Sciences, Wollo University, Dessie, Ethiopia

## Abstract

**Background:**

A drug therapy problem is any undesirable event experienced by a patient during drug therapy that interferes with achieving the desired goals of therapy. It has been pointed out that hospitalized pediatric patients are particularly prone to drug-related problems. Identifying drug therapy problems enables risk quantification and determination of the potential impact of prevention strategies. The purpose of this study was to assess the drug therapy problems in a pediatric ward of Dessie Referral Hospital, northeast of Ethiopia, and to identify associated factors for drug therapy problems.

**Methods:**

A prospective observational study design was carried out to assess drug therapy problems in a pediatric ward of Dessie Referral Hospital from February 1, 2018, to May 30, 2018. Ethical approval was obtained, and informed consent was signed by each study participant's parent before the commencement of the study. All patients admitted to the ward during the study period were included in the study. Data was collected by trained pharmacy staffs through medical record reviews of patients using a prepared standard checklist and semistructured questionnaire. The collected data were cleared and checked every day for completeness and consistency before processing. Data were entered, and descriptive statistical analysis was done using SPSS Version 20 Software. A *P* value of less than 0.05 was considered significant.

**Results:**

The participants' mean age was 2.32 years with the standard deviation (SD) of 0.76 years. Among 81 patients, 71 (87.7%) of them had at least one drug therapy problem per patient which indicates that the prevalence of the drug therapy problem was substantially high. Needs additional drug was the most predominantly encountered drug therapy problem accounted (30 (25.2%)). On the other hand, ineffective drug was the least (3 (2.5%)) drug therapy problem. Antibiotics (47 (39.5%)) followed by fluid and electrolyte (25 (21%)) were classes of drugs mostly involved in the drug therapy problem. The main risk factors reported to the occurrence of the drug therapy problems were prescribing and dose calculation errors.

**Conclusion:**

The present study revealed that majority of the patients had at least one DTP per patient; this indicates that prevalence of DTP was very high in the study area. Needs additional drug therapy followed by noncompliance was the major cause of the occurrence of DTP. Antibiotics were the main class of drugs involved in the drug therapy problem, and among the risk factors assessed, prescribing and dose calculation errors showed statistical significance.

## 1. Background

Based on the definition of Pharmaceutical Care Network Europe (PCNE) [1], a drug-related problem is defined as an undesirable event involving drug therapy that actually or potentially interferes with desired health outcomes and requires professional judgment to resolve through careful assessment of patients, drugs, and disease information to determine the appropriateness of each medication regimen. Drug-related problems are classified into seven categories, which are unnecessary drug therapy, needs additional drug, ineffective drug, dose too low, dose too high, adverse drug reaction, and noncompliance.

Drug therapy problems are associated with prolonged length of stay and increased economic burden and result in an almost 2-fold increased risk of death [2]. DTPs are the dominant reasons for admission. A review of the literature concerning DTPs explored that 28% of all emergency department visits are medication-related, including adverse events, of which 70%-90% are preventable [3, 4]. Some of the DTPs exist at the time of admission, while others appear during treatment in the hospital [4]. Studies that focus on drug-related hospitalization revealed that 5 to 10% of all admissions are drug-related [5]. In Spain, at least 22% discharged patients suffered from actual or potential DRPs [6].

A multicenter study identified that the main drug therapy problems which cause hospital admission were inappropriate techniques (29%), unnecessary drug (25%), and dose too high (11%). The major classes of drug involved in this problem were anti-infective [7].

Ethiopian hospitals consumed about 50% of hospital budget, which are considered to have a high drug budget compared to the population segment. However, very little is known about how drugs are used in hospitals particularly in pediatric patients; as a result, pediatric patients have been still exposed to a number of drug-related problems in Ethiopian hospitals [8]. Even though the problem is serious, findings concerning this issue are scarce not only in Ethiopia but also in Africa and the world. Due to this, pediatric patients are still within exposure to a number of drug-related problems even though they are very important resources of countries, including Ethiopia. There was no study that has been done on drug therapy problems in the study area, Dessie Referral Hospital, northeast Ethiopia.

Pediatrics are especial populations, so they need special attention in their drug therapy. But they have been faced with a number of drug-related problems because data concerning significance of DTP in pediatric patients is limited in the world [9]. In Ethiopia, very little is known about how drugs are used in pediatrics patients and no study had been available on DTPs. This limitation increased the problem and death of pediatrics. Therefore, this study was aimed at assessing drug-related problems that will be used as input for different stakeholders, thereby minimizing the consequences of the problem. Besides, it will be used as a baseline for researchers in similar fields.

## 2. Methods

A prospective observational study was conducted from February 1, 2018, to May 30, 2018, in Dessie Referral Hospital, to assess drug therapy problems in the pediatric ward. The target population for this study was all patients who were admitted to the pediatric ward of Dessie Referral Hospital during the data collection period. All patients admitted to the ward during the study period were included in the study. All patients of either gender aged above 18 years, patients discharged before crosschecking the collected data and attending the outpatient departments of medicine, or those presenting to the hospital emergency room were not recruited. This made the sample size to a convenient sample of 81 patients. A semistructured checklist was employed to collect the required data for this study. The checklist had two parts: part I (case identification) and part II (subjective data, objective data, laboratory results, assessment, prescribed medications, and identified DTPs with justification). Validity of the study was ensured by pretesting the checklist to a sample of patient medical records with similar characteristics. A pretest was performed on five patient medical records in the pediatric ward of DRP. Data was collected by trained pharmacy staffs through medical record reviews of patients using a prepared standard checklist and semistructured questionnaire. The patients were followed till discharge. Within these time intervals, study subjects' symptoms and clinical and laboratory values were recorded to predict adverse drug reactions. The reliability and accuracy of each drug therapy problem were assessed by an independent clinical pharmacist and physician.

The collected data were cleared and checked every day for completeness and consistency before processing. Data were entered, and descriptive statistical analysis was done using SPSS Version 20 Software. A *P* value of less than 0.05 was considered significant.

## 3. Operational Definitions

### 3.1. Unnecessary Drug Therapy

Unnecessary drug therapy is A DTP that occurs when there is no valid medical indication for the drug at the time, or multiple drug products are used when only single-drug therapy is appropriate, or the condition is best treated with nondrug therapy, or the medical problem is caused by drug abuse, alcohol use, or smoking.

### 3.2. Needs Additional Drug Therapy

Needs additional drug therapy is a DTP that occurs when there is a medical condition needing new drug therapy, or preventive therapy is needed to reduce the risk of developing a new condition, or a medical condition requires combination therapy for better efficacy.

### 3.3. Ineffective Drug Therapy

Ineffective drug therapy is a DTP where the drug is not the most effective for the medical problem, or the drug product is not effective for the medical condition, or the condition is refractory to the drug product being used, or the dosage form is inappropriate.

### 3.4. Dosage Too Low

It is a DTP that occurs when the dose is too low to produce the desired outcome, or the dosage interval is too infrequent, or a drug interaction reduces the amount of active drug available, or the duration of therapy is too short.

### 3.5. Dosage Too High

Dosage too high is a DTP where the dose is too high or the dosing frequency is too short or the duration of therapy is too long for the patient, or a drug interaction causes a toxic reaction to the drug product, or the dose was administered too rapidly.

### 3.6. Adverse Drug Reaction

Adverse drug reaction is a DTP where the drug product causes an undesirable reaction that is not dose-related, or a safer drug is needed because of patient risk factors, or a drug interaction causes an undesirable reaction that is not dose-related, or the regimen was administered or changed too rapidly.

### 3.7. Noncompliance

Noncompliance is a DTP that occurs when the patient does not understand the instructions, or the patient prefers not to take or forgets to take the medication, or the cost of the drug product is not affordable for the patient, or the patient cannot swallow or self-administer the medication properly, or the drug product is not available for the patient.

### 3.8. Pediatrics

Pediatrics are those age groups less than 19 years including premature (born before 37 weeks), neonates (from birth to 28 days), infants (1 month to 1 year), children (above 1 year to 12 years), and adolescent (13 to 18 years) [10].

## 4. Results

81 patients fulfilled inclusion criteria and were considered for analysis. Among 81 participants, 36 (44.4%) were children (Table 1). The participants' mean age was 2.32 years with the standard deviation (SD) of 0.76 years. The majority of the respondents had a history of chronic illness (65 (80.2%)). The mean number of diagnosis per patient was 1.56, and the length of hospital stay per patient was 10.36 (Table 2).

### 4.1. Diagnosis and Medication Use Status of Pediatric Patients Admitted in DRH

During the study, 125 diagnoses were identified; from these, severe pneumonia (31 (24.8%)) followed by severe malnutrition (23 (18.4%)) was the leading cases reported in the hospital (Table 3).

Among the total of 311 prescribed drugs, 114 (36.7%) were antibiotics (Table 4). The majority of patients (24 (27.2%)) had a prescription with three drugs. The mean numbers of prescribed drugs were 3.84 per patient ranging from 1 to 8 for minimum and maximum, respectively. This indicates that polypharmacy is a common practice in the hospital.

### 4.2. Types and Number of DRPs Encountered in Pediatric Patients Admitted to DRH

**Figure 1:**
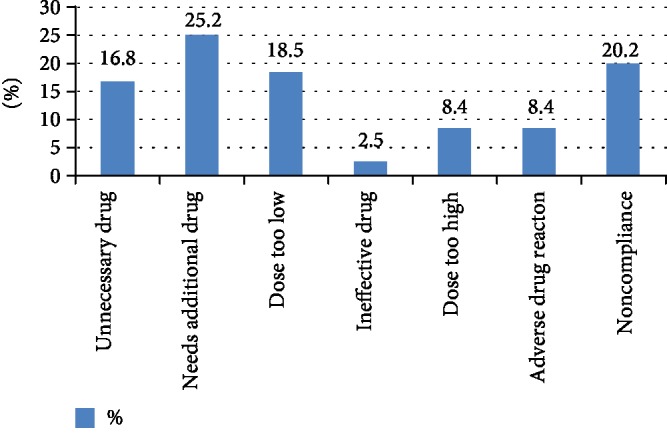
Incidence of DTPs in the pediatric ward of DRH, February 1, 2018, to May 30, 2018.

Among 81 patients, 71 (87.7%) of them had at least one drug-related problem per patient; this indicates that prevalence of the problem is substantially high. A total of 119 DTPs were obtained with a mean of 1.5 drug-related problem per patient ranging from 1 to 4. DTPs were classified into seven along with respective magnitude. Needs additional drug was the most predominantly encountered drug-related problem accounted (30 (25.2%) of the total DTPs). The other common DTPs contained 24 (20.2%), 22 (18.5%), and 20 (16.8%) for noncompliance, dose too low, and unnecessary drug, respectively (Figure 1). Even though most patients were children, majority of the DTPs occurred in infants (58 (48%)) (Table 5). This indicates that infants had higher chance of being affected by DTP. Antibiotics (47 (39.5%)) followed by fluid and electrolyte (25 (21%)) were classes of drugs mostly involved in the drug therapy problem (Table 6).

## 5. Discussion

According to this study, antibiotics were the most frequently prescribed class of drugs. This is because infectious diseases were more common, and also, antibiotics were prescribed as prophylaxis particularly for patients diagnosed with severe malnutrition as these patients are at risk of developing infection even with a single microorganism. The majority of patients (24 (27.2%)) had a prescription with 3 drugs which is comparable to the study by Waltangong et al. [11] with median of 4 drugs per patient. But the maximum numbers of drugs prescribed per patients in Waltangong et al. [11] were 24 which greatly differ from our finding which is 8 drugs per patient, and this can be due to the difference in the prevalence of diseases, existence of comorbidity, and prescribing pattern.

From the total of 81 patients, 71 (87.7%) of them had at least one drug-related problem per patient; this describes that the prevalence of the drug-related problem was substantially high which was comparable to the finding of Waltangong et al. [11]. In this study, a total of 119 DTPs were identified with a mean of 1.5 drug-related problem per patient. Needs additional drug therapy was the leading DTP identified in the study accounting to 25.2%, and this was similar to the study in four French-speaking countries [12] and 5 pediatric clinics in Greece which explained needs additional drug as the most common DTP with 25% and 28%, respectively. Nonadherence was the most common DRP explored in Victoria and Bangkok (50% and 36%, respectively); the same is true in this study as it accounted to 20.2%, next to needs additional drug therapy. This study showed ineffective drug as the least drug-related problem encountered (2.5%); this resembled the study by Aquilina et al. However, the study in a Brazilian hospital stated that this problem was the predominant DTP that contained 46.4% [13]. This variation could be due to the difference in professional diagnosis and drug selection experience. A study in London revealed ADR being the most common DTP obtained (76.6%), but it is less common in our study accounting to 8.4% from all DRPs identified.

In this study, the prescribing problem was the strongest predictor for the occurrence of DRP at *P* < 0.000 (*r* = 0.389) which was comparable to the finding of Waltangong et al. [11]. This may be attributable to missing the prescribed drugs even if there was valuable indication. This can also be described by prescribing drugs for cases that are self-limiting and for those that do not need drug therapy on the time of prescribing and the prescribed medication that is inappropriate for the compiling indication, as all of these conditions cause the occurrence of DTP. However, dosing error which is a strong predictor in our study at *P* < 0.05 (sig = 0.028, *r* = 0.244) was less significant in Waltangong et al. [11]. This variation may be due to nonadherence to standard treatment guidelines to calculate the required dose based on the patients' weight and age. This is also further explained by unavailability of pediatric formulations; thus, the dose is extrapolated from adult counterparts which are unrepresentative of pediatrics leading to causing over- or underdose. Studies in UK and KSA show that the number of prescription and types of admission were potential risk factors for DRPs occurring in children, and studies in London show that prolonged hospitalization, greater number of prescriptions during hospitalization, and dialysis treatment were significant risk factors for higher rates of DRPs. On the other hand, this study revealed that these factors were not significantly associated with the occurrence of DTP.

According to this finding, drug classes involved in DRP were antibiotics (39.5%), fluid and electrolytes (21%), minerals and vitamins (12.6%), and corticosteroids (9.2%). Study in four French-speaking countries also stated that antibiotics were a dominant class of drugs. On the other hand, a study in a Brazilian hospital explored that analgesics were a common class of drugs responsible for DRP. Waltangong et al. [11] in Germany revealed that antiepileptic and corticosteroids were frequently involved in the occurrence of DRP which is comparable to our finding in which corticosteroids were involved.

## 6. Conclusion

The present study revealed that majority of the patients had at least one DTP per patient; this indicates that prevalence of DTP was very high in the study area. Needs additional drug therapy followed by noncompliance was the major causes to the occurrence of DTP. Antibiotics were the main class of drugs involved in the drug therapy problem, and among the risk factors assessed, prescribing and dose calculation errors showed statistical significance.

## Figures and Tables

**Table 1:** Sociodemographic characteristics of patients admitted in DRH, February 1, 2018, to May 30, 2018.

	Frequency	Percent (%)
Age		
Adolescent	11	13.6
Children	36	44.4
Infant	31	38.3
Neonate	3	3.7
Total	81	100.0
Gender		
Female	30	37.0
Male	51	63.0
Total	81	100.0
History of chronic illness		
No	65	80.2
Yes	16	19.8
Total	81	100.0
Past medication history		
No	56	69.1
Yes	25	30.9
Total	81	100.0
Immunization		
Yes	70	86.4
No	11	13.6
Total	81	100.0
Home medication		
No	71	87.7
Yes	10	12.3
Total	81	100.0
Administration error		
No	74	91.4
Yes	7	8.6
Total	81	100.0
Prescribing error		
Yes	39	48.1
No	42	51.9
Total	81	100.0
Omission error		
No	69	85.0
Yes	12	15.0
Total	80	100.0
Dose calculation		
No	56	70.0
Yes	25	30.0
Total	81	100.0
Frequency reduced		
No	80	98.8
Yes	1	1.3
Total	81	100.0
Duration shortened		
No	76	93.8
Yes	5	6.3
Total	81	100.0
Unavailability		
No	75	92.4
Yes	6	7.6
Total	81	100.0

**Table 2:** The mean and standard deviation of continuous independent variables.

	*N*	Minimum	Maximum	Mean	SD
Age of the respondents (year)	81	27/12	14	2.32	0.76
Number of medication per patient	81	1	8	3.84	1.54
Weight of the patient in kilogram	81	3	45	14.93	11.03
Number of diagnosis per patient	81	1	4	1.56	0.67
Length of hospital stay per patient	81	1	30	10.36	7.35

**Table 3:** Diagnosis in the pediatric ward of DRH, February 1, 2018, to May 30, 2018.

Diagnosis	Frequency	Percentage
Severe pneumonia	31	24.8%
Severe malnutrition	23	18.4%
Acute gastroenteritis	16	12.8%
Hyperacute airway disease	13	10.4%
Pyogenic meningitis	9	7.2%
Acute glomerular nephritis	8	6.4%
Sepsis	6	4.8%
Others^∗∗∗^	19	15.2%

Key: others: amoebiasis, nephrotic syndrome, and diabetes mellitus.

**Table 4:** Frequently prescribed classes of drugs in DRH, February 1, 2018, to May 30, 2018.

Classes of drug	Frequency	Percentage
Antibiotics	114	36.8%
Fluid and electrolytes	82	26.4%
Antipyretics	38	12.2%
Minerals and vitamins	24	7.7%
Corticosteroids	21	6.8%
Others^∗^	31	10%

Others^∗^: antiheliments, antiemetic, antiparasites, and insulin.

**Table 5:** Distribution of DTPs in different age groups, February 1, 2018, to May 30, 2018.

Age of the patient	Number	Percentage
Neonates	6	5%
Infants	58	48%
Children	47	39.5%
Adolescent	8	6.7%
Total	119	100%

**Table 6:** Classes of drugs involved in DRTPs, February 1, 2018, to May 30, 2018.

Drug classes	Frequency (percentage)
Antibiotics	47 (37.9%)
Fluid and electrolytes	26 (21%)
Corticosteroids	14 (11.3%)
Vitamins and minerals	13 (10.5%)
Others	24 (19.4%)
Total	124 (100%)

## Data Availability

All the datasets used/or analyzed during the current study are available from the corresponding author on reasonable request.

## Abbreviations

DTP: Drug therapy problem
DRH: Dessie Referral Hospital
ADR: Adverse drug reaction
DDI: Drug-drug interaction
PCNE: Pharmaceutical Care Network Europe
ACE: Angiotensin-converting enzyme
PICU: Pediatric intensive care unit
PEU: Pediatric emergency unit
SAM: Severe acute malnutrition.
